# Rapid increase in transparency of biological organs by matching refractive index of medium to cell membrane using phosphoric acid

**DOI:** 10.1039/c9ra01445d

**Published:** 2019-05-17

**Authors:** Masakazu Umezawa, Shinsuke Haruguchi, Rihito Fukushima, Shota Sekiyama, Masao Kamimura, Kohei Soga

**Affiliations:** Department of Materials Science and Technology, Faculty of Industrial Science and Technology, Tokyo University of Science 6-3-1 Niijuku, Katsushika-ku Tokyo 125-8585 Japan masa-ume@rs.noda.tus.ac.jp mail@ksoga.com; Imaging Frontier Center, Research Institute for Science and Technology (RIST), Organization for Research Advancement, Tokyo University of Science 2641 Yamazaki Noda-shi Chiba 278-8510 Japan

## Abstract

Tissue clearing is a fundamental challenge in biology and medicine to achieve high-resolution optical imaging of tissues deep inside intact organs. The clearing methods reported up to now require long incubation times or physical/electrical pressure to achieve tissue clearing, which is done by matching the refractive indices of the whole sample and medium to that of the lipid layer. Here we show that phosphoric acid increases the refractive index of the medium and can increase the transparency of formalin-fixed tissue samples rapidly. While phosphoric acid (8.5–14.2 M) suppresses bright signals on the boundary of cells in their phase-contrast images, it does not damage the morphology of the phospholipid cell membrane. Immersion of fixed tissues of mice in phosphoric acid solutions (8.5–14.2 M) increased their transparency within 60 min in the case of 3 mm-thick fixed tissue specimens. Although further investigations are needed to apply this protocol to three-dimensional fluorescence imaging or immunohistochemistry, the protocol presented herein may contribute to developing better and faster soaking methods for tissue clearing than previously reported protocols.

## Abstract 2

Tissue clearing effect of phosphoric acid is fast and needs only 60 min incubation.

## Graphical abstract

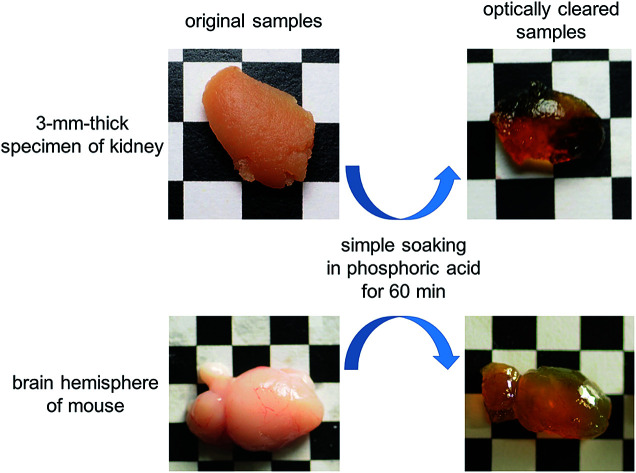

## Introduction

Whole-tissue and whole-body imaging of single cells in opaque tissues has been a fundamental challenge in biology and medicine. Biological three-dimensional (3D) imaging has been achieved by reconstructing structure from images of mechanically sectioned tissues, for example serial block-face scanning electron microscopy^1^ and array tomography.^2^ However, the technique is not only labor-intensive and time-consuming but also error-prone.^3^ The utility of rendering tissue optically transparent is well known for 3D imaging of cell populations located deep inside intact tissue. Optical sectioning provides a potentially fast, simple and inexpensive alternative to 3D reconstruction; however, its use for deep imaging is prevented by tissue opacity.

Optical imaging of thick tissues is mostly limited by scattering of imaging light through thick tissues, which contain various cellular and extracellular structures with different refractive indices. The imaging light traveling through different structures is scattered and loses its excitation and emission efficiency, resulting in reduced resolution and imaging depth.^4^ The low refractive index of water compared with that of cellular structures containing proteins and lipids has been the major issue in this regard; in order to address this problem, the main concept of tissue clearing is to adjust the difference between the refractive index of the medium and that of the lipid bilayer.^5,6^ The clearing was achieved by hydrophobic solutions during the early stage of technical development.^7^ In this method, a fixed sample is incubated in a mixture of benzyl alcohol and benzyl benzoate after dehydration of the sample with ethanol and hexane. Subsequent reports showed that the use of tetrahydrofuran or dibenzyl ether instead of alcohol for dehydration improved the clearing of entire brains of adult mice^8,9^ and this method is applicable to clearing lipid-rich spinal cord.^10^ Removing membrane lipids by repetitive organic solvent-based dehydration–rehydration improved the permeabilization of tissues.^11^

To reduce the denaturation and to improve the retention of fluorescent signals in formalin-fixed tissue samples, urea-based hydrophilic mixtures were developed, called Scale.^12^ Because this technique provided a simple, inexpensive alternative to array tomography and serial section electron microscopy, it contributed to promoting and devising further protocols for tissue clearing. Another protocol, using formamide and formamide/poly(ethylene glycol), called Clear^T^ and Clear^T2^, was able to clear mouse embryo and brain with no detergents or solvents.^13^ A method called SeeDB, an abbreviation for ‘see deep brain’, using fructose and α-thioglycerol, renders tissue samples transparent to allow analyses of cellular morphology without removing any components of tissues during the clearing process and thus has minimum deformation artifacts.^14^ Adding fructose to urea can control urea-mediated tissue expansion during the clearing process.^15^ Sugar and sugar–alcohol solutions also achieve clearing of 100 μm-thick mouse brain slices.^16^ Preparation of clearing solutions to adjust the refractive index of samples to that of immersion oil (1.518) on the objective lens is effective for high-resolution fluorescence imaging.^17^ Electrophoresis to remove lipids after embedding tissue into hydrogel polymer contributes to better tissue clearing in a method named CLARITY (clear lipid-exchanged acrylamide-hybridized rigid imaging/immunostaining/*in situ* hybridization-compatible tissue-hydrogel).^18^ However, because this method was technically complex, a passive tissue clearing approach using different fixative-hydrogel monomer crosslinking solutions was subsequently reported for adult rodents or human post-mortem brain.^19,20^ A solution of 2,2′-thiodiethanol is also effective for clearing brains^21^ and facilitated 3D observation of formalin-fixed and fluorescent-labeled whole mouse brains and 2 mm-thick blocks of human brains in combination with CLARITY.^22^ To improve the methodology to deliver reagents deep inside thick tissues, an active clearing technique, ACT-PRESTO (active clarity technique-pressure related efficient and stable transfer of macromolecules into organs), was reported for rendering large tissue samples such as rat and rabbit brains, and even whole bodies of adult mice, optically transparent.^23^ On the other hand, a method called CUBIC, an abbreviation for ‘clear unobstructed brain imaging cocktails and computational analysis’, achieves tissue transparency by passively clearing phospholipids using a mixture of urea and amino alcohol,^24^ which decolorizes blood by efficiently eluting the heme chromophore from hemoglobin.^25^ Optimized CUBIC protocols for whole-body transparent observation of mice^26^ and organs of rats^27^ have also been reported. To reduce tissue damage and degradation, the use of a mild tissue-permeant sugar–alcohol and sorbitol was reported as a method that allows optical reconstructions of 3D mapping of amyloid plaques, neurons and microglia by successive fluorescence and electron microscopy.^28^ However, one of the major issues is that the protocols reported above for clearing tissues require long incubation times, from several hours to days, or pressure that also damages the samples. Rapid and simple soaking protocols for tissue clearing are thus desirable.

Many hydrophilic solvents for tissue clearing contain molecules of high refractive index to increase the refractive index of the incubation medium, surfactant to delipidate^29^ and potentially to promote the infiltration of the reagent deeper into tissues, and urea.^12,15,24,28,30^ The refractive index of a material is proportional to the square root of the dielectric constant of that material, while the lipid membrane is composed of non-polarized fatty-acid chains and polarized phosphate groups. Therefore, we hypothesized that tissue clearing may be achieved not only by increasing the refractive index of the medium but also by affecting polarization and dielectric coefficient of the lipid membrane, owing to urea in the solutions. In particular, the research group who developed Scale, containing urea, Triton X-100, and glycerin, showed that partial tissue clearing was achieved using only urea and the surfactant.^12^ Even though hydration ability, which accelerates the penetration of water and other chemicals, is proposed as a possible mechanism underlying the tissue clearing ability of urea,^30^ the mechanism of its clearing effect is still important to explore. In the present study, we first investigated the potential contribution of urea to adjusting the refractive indices of the medium and the lipid membrane. Next, the tissue clearing effect of phosphoric acid, an anionic molecule which does not interact with the anionic lipid bilayer but increases the refractive index of the medium, was investigated. We showed that urea does not directly contribute to decreasing the refractive index of the lipid bilayer, and that phosphoric acid, which increases the refractive index of the medium, is able to clear the tissues. Although the incubation for fixed tissue samples in phosphoric acid involves only passive immersion, the incubation time required for clearing 3 mm-thick specimens of organs of mice is shorter (≈60 min) compared with the case of other clearing reagents. Phosphoric acid (14.2 M) also increased the transparency of whole hemispheres of mouse brains within 60 min. The rapid optical clearing protocol using phosphoric acid will contribute to further improvement of tissue clearing protocols and will aid in the advancement of biological research.

## Experimental

### Materials

Urea was purchased from Kanto Chemicals (Tokyo, Japan). Phosphoric acid, sodium iodide, indium chloride, 1,2-dipalmitoyl-*sn*-glycero-3-phosphocholine (DPPC), 1,2-dioleoyl-*sn*-glycero-3-phosphocholine (DOPC), cholesterol, ethanol, chloroform, formaldehyde, and Triton X-100 were purchased from Fujifilm Wako Pure Chemical Co (Osaka, Japan). Phosphate buffered saline (PBS) was purchased from Thermo Fisher Scientific K.K. (MA, USA). *N*-(Carbonyl-methoxypolyethyleneglycol 2000)-1,2-distearoyl-*sn*-glycero-3-phosphoethanolamine (DSPE-PEG; Sunbright DSPE-020CN) was purchased from NOF America Corporation (NY, USA). All the reagents were used without further purification, and handled safely without touching directly, in particular for phosphoric acid of high concentration.

### Measurement of refractive index of solutions

Solutions of saturating concentration of DPPC and 4 M urea were prepared in ethanol. The refractive indices of the mixed solutions in ethanol, water, and aqueous solutions of phosphoric acid (4–14.2 M), urea (4 M), sodium iodide (2 and 4 M), indium chloride (4 M), and ScaleA2 were measured by Abbe's refractometer (NAR-1T, Atago Co., Ltd, Tokyo, Japan).

### Measurement of refractive index and optical loss of liposome suspension

Liposome suspension was prepared by the Bangham method.^31^ Briefly, a mixture of 12 μmol of DPPC, 12 μmol of DOPC, 12 μmol of cholesterol, and 4 μmol of DSPE-PEG was prepared in 1600 μL of chloroform in an eggplant-shaped glass flask, and then evaporated at 37 °C to remove chloroform and to obtain a thin lipid bilayer on the inner wall of the flask. 7.5 mL of either distilled water, aqueous solution of urea (4 M) or phosphoric acid (14.2 M) was added into the flask and sonicated to prepare a liposome suspension in each liquid. The optical loss (OD600) of the liposome suspensions was measured by using a V-660 spectrometer (JASCO Co., Tokyo, Japan) and a 1.5 mL disposable cuvette (Brand GmbH & Co. KG, Wertheim, Germany).

### Measurement of optical loss of cell suspension of mouse brain

All animals were handled in accordance with the Japanese national guidelines for the care and use of laboratory animals and with the approval of the Animal Care and Use Committee of the Tokyo University of Science. Brain samples isolated from adult ICR mice sacrificed under hyper-anesthesia were minced by using a BioMasher (Nippi Inc., Tokyo, Japan), suspended in PBS, and fixed using 4 wt% formaldehyde in PBS overnight. Fixed cell suspension was collected by centrifugation (1200 × *g*, 5 min), washed with PBS three times, and divided into different tubes. The cell suspensions were then incubated in either ScaleA2, PBS, or an aqueous solution of urea (4 M), indium chloride (4 M), sodium iodide (2, 4 M), Triton X-100 (10 wt%), phosphoric acid (8.5, 11.4, 14.2 M), or 0.1 wt% Triton X-100 in phosphoric acid (14.2 M) for 24 h at room temperature. The OD600 was measured by means of a V-660 spectrometer (JASCO) and a 1.5 mL disposable cuvette (Brand GmbH & Co. KG, Wertheim, Germany).

### Observation of morphology and phase-contrast image of formalin-fixed cells

Cultured Colon-26 cells were maintained in Minimum Essential Medium [(+)Eagle's salt, (+)l-glutamine] (Gibco, Thermo Scientific, MA, USA) supplemented with 10% fetal bovine serum (Biowest, Nuaillé, France) and 1% penicillin–streptomycin (Wako, Tokyo, Japan) at 37 °C in humidified air containing 5% CO_2_. After staining of the cell membrane by CellMask Orange (Thermo Fisher Scientific), the cells were fixed using 4 wt% formaldehyde in PBS for 10 min, washed with PBS, and then incubated in phosphoric acid (14.2 M) for 0–48 h. Cell membrane stained with CellMask was observed by fluorescence microscopy through a band-pass filter (cut-on wavelength: 575 nm) under excitation (540 ± 10 nm). Unstained cells were also fixed with formalin, washed with PBS, incubated in ScaleA2, phosphoric acid (14.2 M), or PBS for 24 h, and observed under phase-contrast microscopy.

### Measurement of optical loss of mouse tissues

Brain, liver, kidney, and lung samples were obtained from adult ICR mice after blood removal from the aortas under anesthesia. The samples of liver, kidney, and lung were fixed with 4 wt% formaldehyde in PBS for 48 h, washed with PBS, and cut into approximately 3 mm-thick specimens. The fixed tissue specimens and brain hemispheres of mice were incubated in either ScaleA2, PBS, or an aqueous solution of indium chloride (4 M), sodium iodide (4 M), Triton X-100 (10 wt%), or phosphoric acid (8.5, 11.4, 14.2 M) for 60 min. The macroscopic images of the treated samples were captured by a digital camera. To confirm the reversibility of tissue clearing, 450 μm-thick specimens of mouse brain were prepared, fixed with formaldehyde for 60 min, washed with PBS, incubated in 14.2 M phosphoric acid or PBS for 60 min, replaced and kept in PBS for 24 h, and their images were also recorded by digital camera.

## Results and discussion

**Fig. 1:**
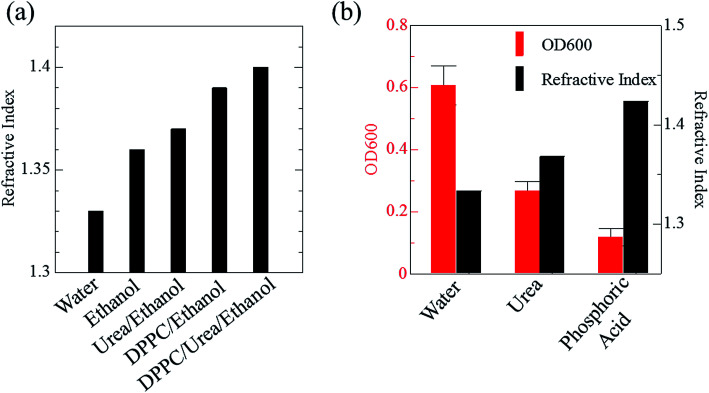
Effects of urea and phosphoric acid on the refractive indices of solvents and on transparency of liposome suspension. Refractive indices of DPPC in ethanol and its mixture with urea are shown in (a). An increase in the refractive index of DPPC solution in ethanol due to urea suggests that urea does not influence the polarization of the phosphoric acid groups of the lipid bilayer but increases the refractive index of the medium. The effects of urea (4 M) and phosphoric acid (14.2 M) on the refractive index of the medium and optical loss of liposome suspension at 600 nm (OD600) are shown in (b). The OD600 data are shown as means ± SD (*n* = 3). Abbreviation: DPPC, 1,2-dipalmitoyl-*sn*-glycero-3-phosphocholine.

Phospholipid, containing both hydrophobic and hydrophilic parts, is arranged in three layers in the plasma membrane; the hydrophobic part is in the interior of the membrane, whereas the hydrophilic part points outwards, toward either the cytoplasm or the fluid that surrounds the cell. The hydrophobic and hydrophilic parts have different refractive indices owing to the difference in their dielectric constants. Our hypothesis was a possible contribution of decrease in polarization of the phosphate group, induced by electrostatic interaction of the polarized group with urea, to decreasing the refractive index difference between the lipid bilayer and medium. The refractive index of the solution of a representative phospholipid, dipalmitoylphosphatidylcholine (DPPC), in ethanol, which is able to dissolve both DPPC and urea, was higher than those of ethanol and water (Fig. 1a). Adding urea to ethanol and to DPPC solution in ethanol increased their refractive indices (Fig. 1a). These results suggest that urea does not decrease the refractive index of DPPC in ethanol but increases the refractive indices of the solutions. The optical loss of liposomes suspended in an aqueous solution of urea (4 M), which shows higher refractive index than water, was higher than that of liposomes suspended in water (Fig. 1b). Moreover, 14.2 M phosphoric acid, which is an anionic molecule that does not interact with the anionic phosphate group of lipid bilayers, shows higher refractive index than 4 M urea solution and decreased the optical loss of the liposomes (Fig. 1b). We concluded that urea increased the transparency of liposomes not by affecting the polarization or refractive index of phosphate groups of the lipid bilayer, but by reducing the difference in refractive indices between the medium and the lipid bilayer, as did phosphoric acid. The clearing effect of phosphoric acid is greater than that of 4 M urea owing to its higher refractive index.

**Fig. 2:**
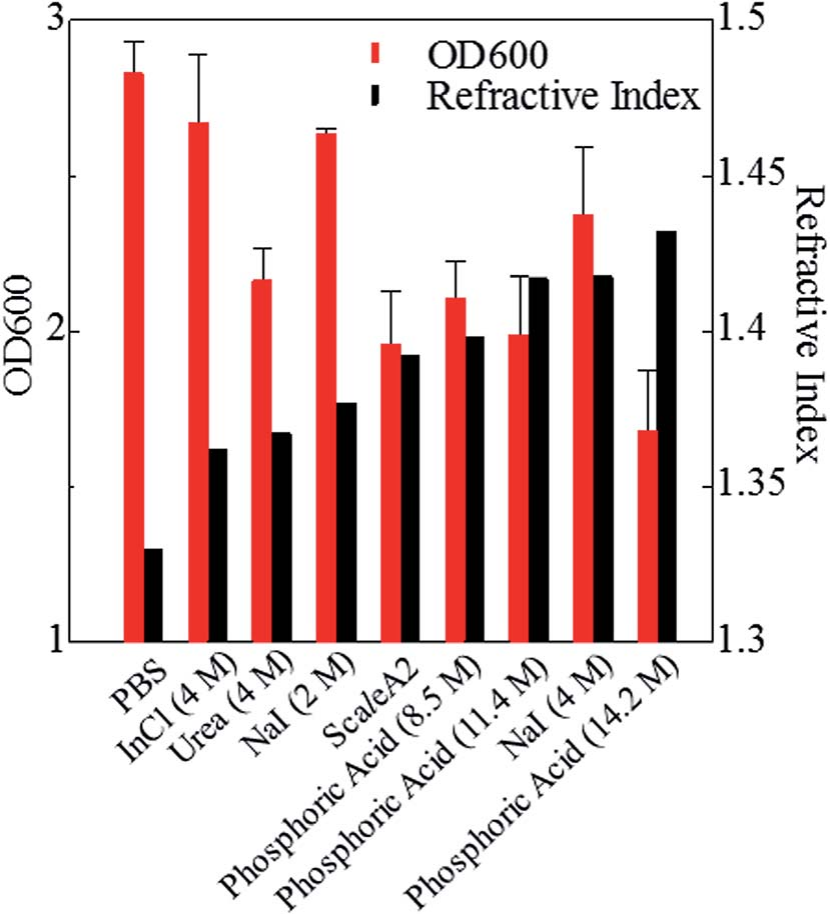
Effects of candidate solutions with different refractive indices on light loss for fixed cell suspension. Effects of 24 h incubation in candidate chemical solutions with various refractive indices on OD600 values (means ± SD, *n* = 3) of brain cell suspension fixed with formaldehyde are shown. Phosphoric acid (8.5, 11.4, 14.2 M) further increases the refractive index of the medium and the transparency of the cell suspension compared with sodium iodide (4 M), urea (4 M), and indium chloride (4 M). Abbreviations: InCl, indium chloride; NaI, sodium iodide; PBS, phosphate buffered saline.

**Fig. 3:**
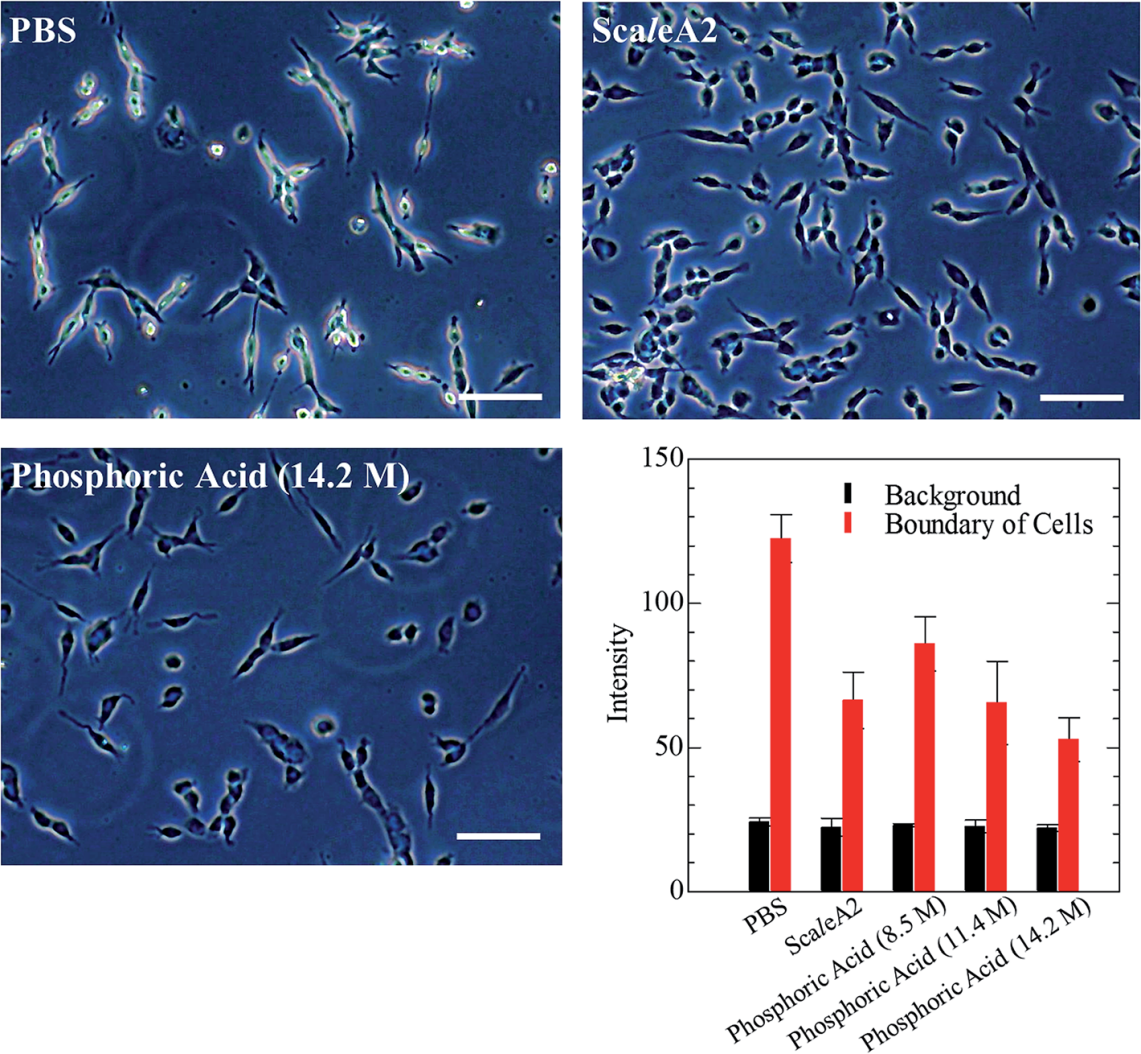
Phase-contrast images of formalin-fixed cultured colon-26 cells incubated in phosphoric acid or ScaleA2. Effect of ScaleA2 and phosphoric acid (14.2 M) solution on the phase contrast at the edge of cultured cells is shown in the images. Scale bars indicate 50 μm. The graph shows the concentration-dependent effect of phosphoric acid (8.5–14.2 M) on the gray values of background and the edge in the microscope images.

**Fig. 4:**
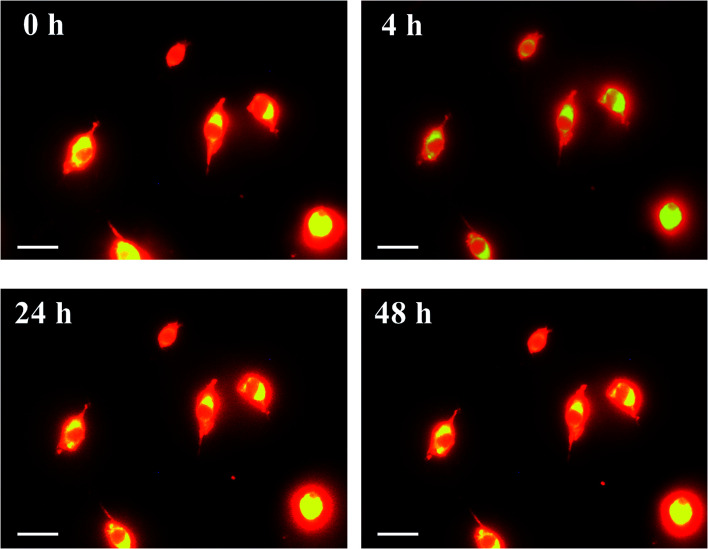
Images of formalin-fixed plasma membrane of cultured cells incubated in phosphoric acid. Cultured colon-26 cells were stained with CellMask Orange (Thermo Fisher Scientific), fixed by using 4 wt% formaldehyde in PBS, washed with PBS, and then incubated in phosphoric acid (14.2 M) for 0–48 h and observed under a microscope. Scale bars indicate 10 μm. The images confirmed that formalin-fixed cell membrane was retained during incubation in the solution.

**Fig. 5:**
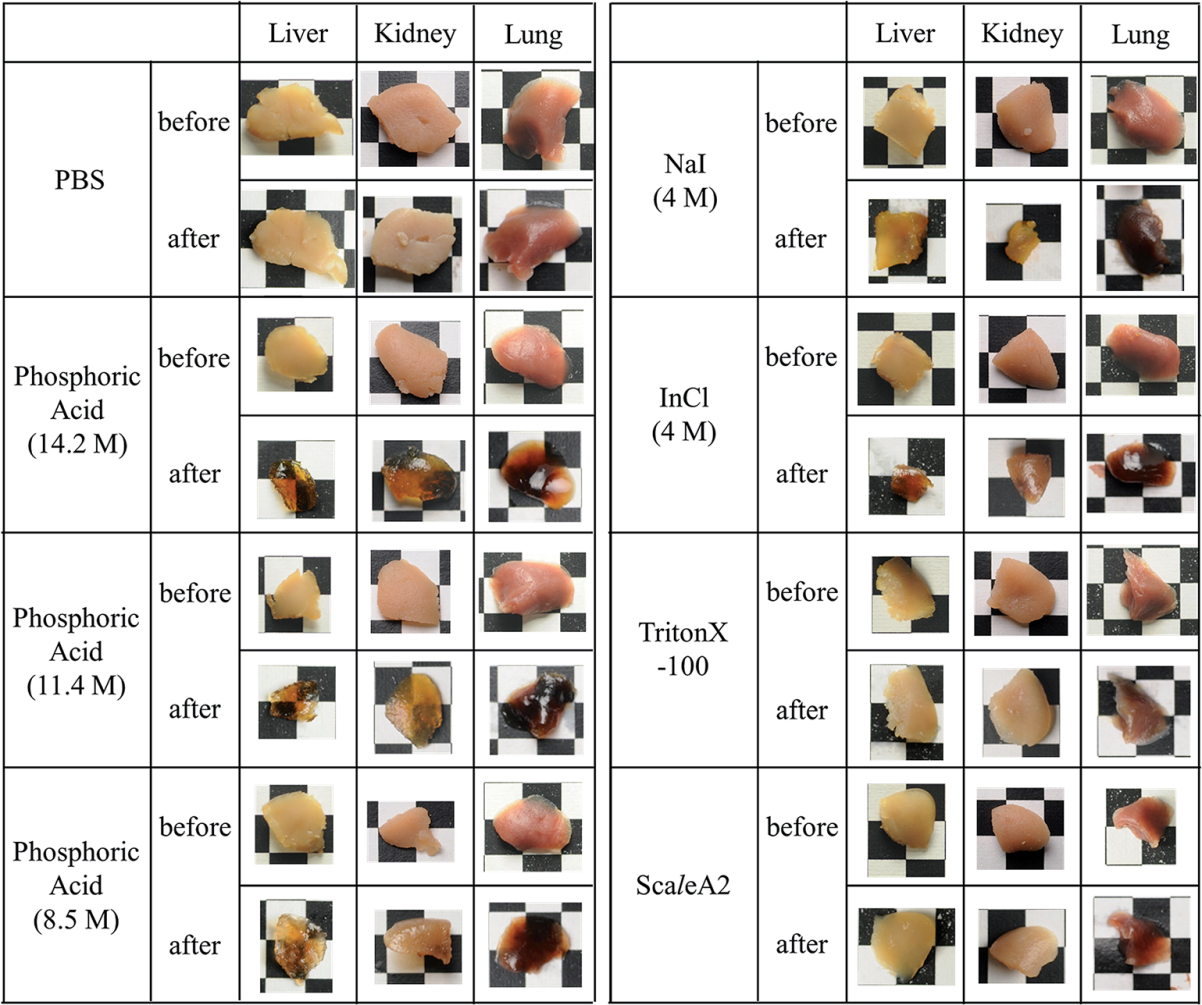
Effect of incubation in chemical solutions on macroscopic images of formalin-fixed tissue specimens from mice. The specimens of liver, kidney, and lung of mice were fixed with 4 wt% formaldehyde for 48 h, washed with PBS, and then incubated in the indicated chemical solutions for 60 min. The apertures of the background lattice are 4 mm.

**Fig. 6:**
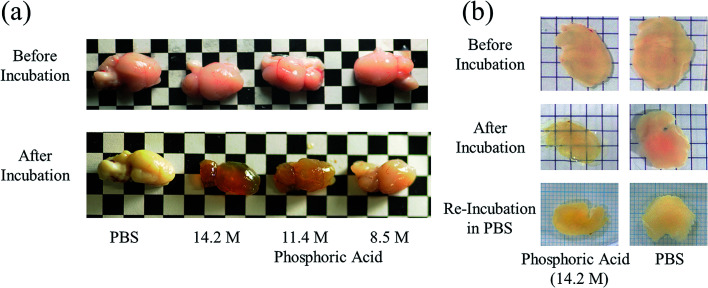
Clearing mouse brains by means of phosphoric acid. Representative images of (a) hemispheres and (b) 450 μm-thick specimens of mouse brains before and after 60 min incubation in phosphoric acid (8.5, 11.4, and 14.2 M) are shown. As shown in (b), brain tissue that had been cleared once by using phosphoric acid (14.2 M) returned to the opaque state after re-incubation in PBS for 24 h.

Next, we investigated the clearing effect of phosphoric acid, which decreased the light loss of the liposomes, and other candidate chemical solutions on fixed cell suspensions of mouse brain tissues. We targeted phosphoric acid solution because the phosphate group is strongly polarized and thus contributes to the high refractive index of the phospholipid bilayer. Tissue clearing effects of many kinds of solvents were evaluated by measuring the light loss (OD600) of suspensions of tissue cells fixed with formaldehyde after homogenization, as in previous studies.^24^ The loss of light intensity by formalin-fixed cell suspensions of mouse brain was decreased by 24 h incubation in phosphoric acid and ScaleA2 (Fig. 2). On the other hand, although sodium iodide (4 M) solution has a similar high refractive index to phosphoric acid (11.4 M), sodium iodide did not decrease the light loss of the cell suspension during the 24 h incubation. Indium chloride (4 M) also has a similar refractive index to urea (4 M); it did not attenuate the light loss due to the 24 h incubation as well as sodium iodide. Incubation of cultured cells in tissue clearing solutions (phosphoric acid and ScaleA2) attenuated the bright signal on the boundary of cells in the phase-contrast image in a concentration-dependent manner (Fig. 3). Because the bright signal on the cell boundary is generated where the difference in refractive index between the sample and background is large, the decrease in the signal caused by the clearing solutions supports the effects of the solutions on the refractive index difference between the cell membrane and medium. Care should be taken regarding the possibility that the contrast in the phase-contrast image may be also affected by cell-substrate attachment. The stability of formalin-fixed cell membrane in phosphoric acid was confirmed by labeling the membrane of the cultured cells, suggesting that phosphoric acid does not damage the fixed membrane during incubation (Fig. 4). Macroscopic images of formalin-fixed organ specimens (liver, kidney, and lung) of mice demonstrated that the samples were partially cleared by 60 min incubation in phosphoric acid solution (Fig. 5). The visibility of a black and white checker board through the tissues was evaluated after incubation (Fig. 5). The results showed that phosphoric acid (8.5 M) is effective for reducing scattering by the liver, while higher concentrations of 11.4 M and 14.2 M are needed for the kidney and lung, respectively. The samples were not damaged by incubation in phosphoric acid for 60 min. The possibility that the sample is damaged by longer (*e.g.*, >24 h) incubation should be taken into consideration. The white scattering by tissues was reduced, while the color remained after the incubation; therefore, the dark-red color looks partially enhanced in the pictures (Fig. 5). Further investigations are required for more successful tissue clearing by combination with decolorization reagents such as aminoalcohol.^25^ The phosphoric acid solutions reduced the scattering within 10 min, while the clearing effect was saturated after 60 min incubation (unpublished data). On the other hand, 60 min incubation was too short to clear the tissue specimens by means of ScaleA2. Rapid tissue clearing by phosphoric acid is possibly due to fast infiltration of the molecule because of its small size. The molecular size of phosphoric acid (PO_4_^3−^) is smaller than that of urea (CH_3_COCH_3_), glycerol [CH_2_(OH)–CH(OH)–CH_2_(OH)], and sugars and sugar–alcohols. Other candidate chemicals investigated in the present study, sodium iodide (4 M) and indium chloride (4 M), did not make the tissues transparent, possibly owing to their lower refractive indices, but induced shrinkage of samples, especially the kidney specimen. Phosphoric acid is also able to make brain hemispheres transparent, as shown in Fig. 6. The clearing by phosphoric acid is reversible: re-incubation of transparent 450 μm-thick brain slices in PBS made the samples as opaque as before phosphoric acid treatment (Fig. 6b). This result indicates that phosphoric acid reduces light scattering in the tissue sample not by irreversible chemical modification but by adjusting the refractive index of the sample to a value close to that of the cell membrane by simple infiltration.

This article just focused on rapid reduction of light scattering of biological tissues by soaking them in phosphoric acid, a small molecule with high refractive index. Because, in scientific research, most tissues need to be analyzed by immunohistochemistry or fluorescence imaging after optical clearing, further experiments are currently underway to investigate the availability of phosphoric salt solutions with less acidity for optical imaging of inside of the cleared tissues.

## Conclusions

The present study showed that phosphoric acid, a hydrophilic solvent, can clear tissues including brain, liver, kidney, and lung of mice. Phosphoric acid reduces light scattering by mouse tissues rapidly, requiring only 60 min incubation, while it does not damage the morphology of the phospholipid cell membrane. Combination of decoloring agents with small molecular solutes that increase refractive index, like phosphoric acid, may achieve better and rapid tissue clearing. The potential of phosphoric salts with neutral pH for tissue clearing is also of future interest because acidic conditions generally denature fluorescent proteins.^32,33^ The rapid reducing of scattering with the use of phosphoric acid as presented here will contribute to developing better and faster soaking methods for tissue clearing than previously reported protocols.

## Conflicts of interest

There are no conflicts of interest to declare.

## Supplementary Material

## References

[cit1] Denk W., Horstmann H. (2004). Serial block-face scanning electron microscopy to reconstruct three-dimensional tissue nanostructure. PLoS Biol..

[cit2] Micheva K. D., Smith S. J. (2007). Array tomography: a new tool for imaging the molecular architecture and ultrastructure of neural circuits. Neuron.

[cit3] Helmstaedter M., Briggman K. L., Denk W. (2008). 3D structural imaging of the brain with photons and electrons. Curr. Opin. Neurobiol..

[cit4] Boas D. (1997). A fundamental limitation of linearized algorithms for diffuse optical tomography. Opt. Express.

[cit5] Genina E. A., Bashkatov A. N., Tuchin V. V. (2010). Tissue optical immersion clearing. Expert Rev. Med. Devices.

[cit6] Richardson D. S., Lichtman J. W. (2015). Clarifying Tissue Clearing. Cell.

[cit7] Dodt H. U., Leischner U., Schierloh A., Jährling N., Mauch C. P., Deininger K. (2007). *et al.*, Ultramicroscopy: three-dimensional visualization of neuronal networks in the whole mouse brain. Nat. Methods.

[cit8] Becker K., Jährling N., Saghafi S., Weiler R., Dodt H. U. (2012). Chemical clearing and dehydration of GFP expressing mouse brains. PLoS One.

[cit9] Ertürk A., Mauch C. P., Hellal F., Förstner F., Keck T., Becker K. (2012). *et al.*, Three-dimensional imaging of the unsectioned adult spinal cord to assess axon regeneration and glial responses after injury. Nat. Med..

[cit10] Ertürk A., Becker K., Jährling N., Mauch C. P., Hojer C. D., Egen J. G. (2012). *et al.*, Three-dimensional imaging of solvent-cleared organs using 3DISCO. Nat. Protoc..

[cit11] Renier N., Wu Z., Simon D. J., Yang J., Ariel P., Tessier-Lavigne M. (2014). iDISCO: a simple, rapid method to immunolabel large tissue samples for volume imaging. Cell.

[cit12] Hama H., Kurokawa H., Kawano H., Ando R., Shimogori T., Noda H. (2011). *et al.*, Scale: a chemical approach for fluorescence imaging and reconstruction of transparent mouse brain. Nat. Neurosci..

[cit13] Kuwajima T., Sitko A. A., Bhansali P., Jurgens C., Guido W., Mason C. (2013). ClearT: a detergent- and solvent-free clearing method for neuronal and non-neuronal tissue. Development.

[cit14] Ke M. T., Fujimoto S., Imai T. (2013). SeeDB: a simple and morphology-preserving optical clearing agent for neuronal circuit reconstruction. Nat. Neurosci..

[cit15] Hou B., Zhang D., Zhao S., Wei M., Yang Z., Wang S. (2015). *et al.*, Scalable and DiI-compatible optical clearance of the mammalian brain. Front. Neuroanat..

[cit16] Yu T., Qi Y., Wang J., Feng W., Xu J., Zhu J. (2016). Rapid and prodium iodide-compatible optical clearing method for brain tissue based on sugar/sugar-alcohol. J. Biomed. Opt..

[cit17] Ke M. T., Nakai Y., Fujimoto S., Takayama R., Yoshida S., Kitajima T. S. (2016). *et al.*, Super-resolution mapping of neuronal circuitry with an index-optimized clearing agent. Cell Rep..

[cit18] Chung K., Wallace J., Kim S. Y., Kalyanasundaram S., Andalman A. S., Davidson T. J. (2013). *et al.*, Structural and molecular interrogation of intact biological systems. Nature.

[cit19] Tomer R., Ye L., Hsueh B., Deisseroth K. (2014). Advanced CLARITY for rapid and high-resolution imaging of intact tissues. Nat. Protoc..

[cit20] Zheng H., Rinaman L. (2016). Simplified CLARITY for visualizing immunofluorescence labeling in the developing rat brain. Brain Struct. Funct..

[cit21] Aoyagi Y., Kawakami R., Osanai H., Hibi T., Nemoto T. (2015). A rapid optical clearing protocol using 2,2'-thiodiethanol for microscopic observation of fixed mouse brain. PLoS One.

[cit22] Costantini I., Ghobril J. P., Di Giovanna A. P., Allegra Mascaro A. L., Silvestri L., Müllenbroich M. C. (2015). *et al.*, A versatile clearing agent for multi-modal brain imaging. Sci. Rep..

[cit23] Lee E., Choi J., Jo Y., Kim J. Y., Jang Y. J., Lee H. M. (2016). *et al.*, ACT-PRESTO: rapid and consistent tissue clearing and labeling method for 3-dimensional (3D) imaging. Sci. Rep..

[cit24] Susaki E. A., Tainaka K., Perrin D., Kishino F., Tawara T., Watanabe T. M. (2014). *et al.*, Whole-brain imaging with single-cell resolution using chemical cocktails and computational analysis. Cell.

[cit25] Tainaka K., Kubota S. I., Suyama T. Q., Susaki E. A., Perrin D., Ukai-Tadenuma M. (2014). *et al.*, Whole-body imaging with single-cell resolution by tissue decolorization. Cell.

[cit26] Kubota S. I., Takahashi K., Nishida J., Morishita Y., Ehata S., Tainaka K. (2017). *et al.*, Whole-body profiling of cancer metastasis with single-cell resolution. Cell Rep..

[cit27] Matryba P., Bozycki L., Pawłowska M., Kaczmarek L., Stefaniuk M. (2018). Optimized perfusion-based CUBIC protocol for the efficient whole-body clearing and imaging of rat organs. J. Biophot..

[cit28] Hama H., Hioki H., Namiki K., Hoshida T., Kurokawa H., Ishidate F. (2015). *et al.*, ScaleS: an optical clearing palette for biological imaging. Nat. Neurosci..

[cit29] Tainaka K., Murakami T. C., Susaki E. A., Shimizu C., Saito R., Takahashi K. (2018). *et al.*, Chemical landscape for tissue clearing based on hydrophilic reagents. Cell Rep..

[cit30] Susaki E. A., Ueda H. R. (2016). Whole-body and whole-organ clearing and imaging techniques with single-cell resolution: toward organism-level systems biology in mammals. Cell Chem. Biol..

[cit31] Bangham A. (1978). Properties and uses of lipid vesicles: an overview. Ann. N. Y. Acad. Sci..

[cit32] Enoki S., Saeki K., Maki K., Kuwajima K. (2004). Acid denaturation and refolding of green fluorescent protein. Biochemistry.

[cit33] Schwarz M. K., Scherbarth A., Sprengel R., Engelhardt J., Theer P., Giese G. (2015). Fluorescent-protein stabilization and high-resolution imaging of cleared, intact mouse brains. PLoS One.

